# *Announcement:* National Arthritis Awareness Month — May 2017

**DOI:** 10.15585/mmwr.mm6620a6

**Published:** 2017-05-26

May is National Arthritis Awareness Month. In the United States, approximately 54 million (*1*) adults have some form of doctor-diagnosed arthritis; this number is projected to increase to 78 million by 2040 (*2*). In addition, arthritis-attributable activity limitation currently affects an estimated 24 million adults with arthritis (*1*), and this is expected to rise to 35 million by 2040 (*2*). Approximately one in three adults with arthritis have severe joint pain (*1*). Arthritis is a leading cause of disability and makes it harder to manage other co-occurring conditions, such as diabetes, heart disease, and obesity. A Vital Signs report on arthritis prevalence (*1*) was published in *MMWR* in March 2017 to increase awareness of arthritis, its impact in the United States, and what can be done by health care providers, adults with arthritis, and state and community leaders to address these issues.

CDC recommends physical activity and a variety of evidence-based physical activity and self-management education programs. Physical activity can reduce arthritis pain and improve function by about 40%, and self-management education workshops, such as the Chronic Disease Self-Management Program, have shown 10%–20% improvements in pain, fatigue, and depression in adults with arthritis (*3*). CDC funds 12 state health departments and several national organizations to disseminate these programs in local communities. Additional information is available at https://www.cdc.gov/arthritis/interventions/index.htm and https://www.cdc.gov/arthritis/partners/index.htm.
